# Ceftriaxone-induced encephalopathy in a patient with a normal renal function

**DOI:** 10.1136/bcr-2023-256934

**Published:** 2024-01-11

**Authors:** Audrius Zarauskas, Bruno Rodrigues, Vincent Alvarez

**Affiliations:** 1 Internal Medicine, Hopital de Sion Centre Hospitalier du Valais Romand, Sion, Switzerland; 2 Neurology, Hôpital de Sion Centre Hospitalier du Valais Romand, Sion, Switzerland

**Keywords:** Neurology (drugs and medicines), Safety, Unwanted effects / adverse reactions

## Abstract

Ceftriaxone-induced encephalopathy is an exceptionally rare adverse effect of this commonly used cephalosporin and is generally observed in patients undergoing haemodialysis or suffering from severe renal failure. We present a case of a fit woman in her mid-80s with a normal renal function who developed severe fluctuating neurological symptoms (aphasia, loss of contact, chorea-like tongue movements) while being treated with ceftriaxone for a urinary tract infection with bacteraemia. The symptoms began on day 4 of treatment and an adverse drug reaction was suspected on day 7, after exhaustive investigations failed to reveal another cause. A complete recovery was observed 3 days after discontinuing ceftriaxone. Our case highlights the need to consider the diagnosis of ceftriaxone encephalopathy, even if the traditional risk factors are lacking. In this article, we also provide a brief overview of the pathophysiology as well as a literature review concerning the subject.

## Background

Ceftriaxone-related adverse neurological effects are uncommon to very rare and mostly mild (dizziness, headache, ataxia, paresthesia).^1^ However, multiple case reports^2–21^ and a case series from a French pharmacovigilance database^22^ have been published on more severe reactions, notably encephalopathy, trouble of consciousness and choreoathetosis. With ceftriaxone being one of the most commonly used parenteral antibiotics worldwide^23^, there is a possibility that many more cases of these severe adverse reactions are not readily recognised or may be confused with common delirium. The vast majority of cases involve patients with either severely impaired renal function or patients on haemodialysis. We present a case of a patient in her mid-80s with a normal renal function who developed severe encephalopathy while being treated with ceftriaxone for a urinary tract infection.

## Case presentation

A fit and autonomous female patient in her mid-80s, known for arterial hypertension, was admitted to our department for pyelonephritis with progressive asthenia and urinary symptoms. The clinical examination was unremarkable except for moderate dehydration and a fever of 39.2°C. Ceftriaxone was started at 2 g/day with a resolution of fever and urinary complaints over the next 2 days.

On day 4, the patient began presenting fluctuating and progressively worsening neurological symptoms, starting out with transitory disorientation and incomprehensible speech. On day 5, we observed fluctuating aphasia and three short episodes of loss of contact, as well as brief myoclonic movements of low amplitude in the four extremities. The symptoms reached their peak on day 7 with the aphasia periods lasting up to 1 hour, the patient gripping and not releasing objects and having chorea-like tongue movements.

## Investigations

The laboratory work at the emergency room showed a leucocytosis (12 x10ˆ9/L), elevated C-reactive protein (122 mg/L), mild hyponatraemia (130 mmol/L) and a normal renal function (creatinine 73 µmol/L, Chronic Kidney Disease Epidemiology Collaboration (CKD-EPI) 64 mL/min/1.73 m^2^). We observed a slight elevation of the creatinine on day 1 (creatinine 88 µmol/L, CKD-EPI 51 mL/min/1.73 m^2^), without the criteria for an acute kidney injury. The renal function normalised the day after with intravenous fluids and stayed stable during the entire hospitalisation afterwards (creatinine 54–66 µmol/L, CKD-EPI 72–82 mL/min/1.73 m^2^).

On day 4 (the beginning of the neurological symptoms), the blood tests showed a normal blood glucose, a stable sodium (130 mmol/L), a slight hypophosphataemia (0.69 mmol/L), normal liver enzymes, liver function tests and coagulation panel. A bladder ultrasound revealed a urinary retention, which was catheterised. No acute postrenal kidney injury was detected. The neurological examination was non-focal, with no signs of meningitis, and the cerebral angio-CT was normal except for mild atrophy.

**Figure 1:**
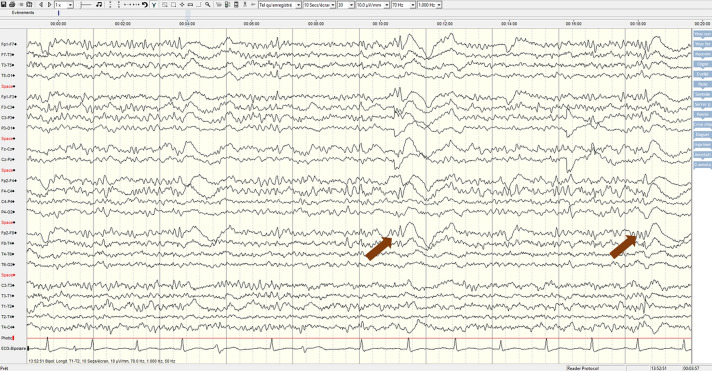
EEG showing an unstable background activity with superimposed diffuse fast activity (beta rhythm). Arrows show examples of generalised discharges with triphasic morphology. Recorded according 10–20 system, high pass filter set at 1 Hz, low-pass filter set at 70 Hz, notch filter set at 50 Hz. Display with a bipolar longitudinal montage, 10 s/pages and 70 µV/cm. EEG, electroencephalogram.

On day 7, in the context of persistent neurological symptoms, we obtained a lumbar puncture (normal, clear cerebrospinal fluid (CSF), 1 erythrocyte/µL, 1 leucocyte/µL, normal glucose and lactate), brain MRI (cortico subcortical atrophy with parietal predominance, no acute changes) and an electroencephalogram (EEG) (figure 1), which showed unstable background activity with superimposed diffuse fast activity (beta rhythm) and generalised discharges with triphasic morphology related to an encephalopathy.

## Differential diagnosis

On the day of the apparition of neurological disturbances, we excluded an acute intracranial bleed, stroke and major electrolyte perturbations and suspected a peri-infectious delirium as the cause for the symptoms.

With the apparition of aphasia and myoclonic jerks on day 5, a state of non-convulsive status epilepticus was suspected. Clonazepam (0.5 mg intravenously) was administered empirically, as a diagnostic test and a therapeutic measure, allowing for a complete resolution of symptoms for a period of about 12 hours.

On the return of symptoms on day 6 and their worsening on day 7, we searched for, and excluded, focal brain lesions, meningo-encephalitis, and epileptic disease. Despite the recent EEG, a non-convulsive status was suspected again by the on-call resident, but this time an administration of clonazepam (0.5 mg intravenously×2) improved symptoms only partially and haloperidol (0.5 mg subcutaneously) had no effect.

We did not measure serum ammonaemia in our patient with normal liver tests, no past hepatic pathology history and absence of asterixis. Rare cases of hyperammonaemia in obstructive urinary tract infections have been described^24 25^ but were marked by a significant and rapid improvement in the neurological status after bladder catheterisation,^24^ on the contrary to our patient whose neurological status continued to decline.

## Treatment

Antibiotic treatment by ceftriaxone was maintained initially because of the worsening neurological condition, despite the blood and urine cultures revealing a multisensitive *Escherichia coli* and the favourable clinical evolution of the urinary infection the days prior. After reasonably excluding structural brain lesions, a central nervous system (CNS) infection, epileptiform activity and electrolyte perturbations, we finally attributed the clinical symptoms to ceftriaxone-induced encephalopathy on day 7. The medication was discontinued, with a switch to amoxicillin-clavulanic acid (intravenous first and per os later).

## Outcome and follow-up

The aphasia symptoms disappeared on day 9 and complete recovery was noted on day 10, 3 days after stopping ceftriaxone. Afterwards, the patient signalled vaguely remembering being asked questions by the staff, but not being able to produce responses. She was discharged home on day 19. A phone call 3 months later confirmed the absence of neurological symptoms or any other general complaints.

## Discussion

We did a literature review on PubMed in April 2023 without a time limit and identified 15 case reports,^4 6–9 11–13 15–18 20 21 26^ 7 small case series^2 3 5 10 14 19 27^ and 2 pharmacovigilance database analysis^22 28^ discussing serious neurological effects of ceftriaxone in humans.

The authors of the French pharmacovigilance database paper found 152 serious reports, with the declared patients presenting mostly with encephalopathy (20.8% of the adverse reactions), confused state (15.7%) and convulsions (13%).^22^ The median onset time was 4 days and the median duration of symptoms was 4.5 days.^22^ Most of the patients were over 65 years old (69.7%) and had renal impairment (74.6%).^22^ The analysis of the Japanese Adverse Drug Event Report database suggested that ceftriaxone might be associated with encephalopathy, and the risk might be increased in patients who have CKD, are receiving ceftriaxone at a dosage of >2 g/day, are treated for more than 14 days or are females.^28^

Table 1 summarises the case reports and the case series. Out of the total 27 patients, 74.1% were 65 years of age or older, 59.3% were females and only 2 (7.4%) had a normal renal function. The median time to onset was 5 days (minimum 2 days, maximum 23 days) and the median time to resolution was 3 days (minimum 1 day, maximum 12 days).

**Table 1:** Summary of cases

Case	Sex/age	Renal function (µmol/L; mL/min/1.73 m²)	Dose (g/day)	Neurological manifestations	Electroencephalogram findings	Days to onset/remission	Treatment	Ref.
1	F 78	CKD (creatinine 457.6, CKD-EPI 8)	2	Drowsiness, myoclonus, single generalised convulsive seizure	NCSE	6/5	↕ CTX (day not specified), clonazepam, sodium valproate	2
2	F 83	AKI (creatinine 176, CKD-EPI 24)	2	Drowsiness, myoclonus	NCSE	4/5	↕ CTX (day not specified), clonazepam, phenytoin, sodium valproate	2
3a	F 12	CPD	100 mg/kg	Confusion, visual hallucinations, facial myoclonus	Generalised spike and spike-wave discharges, generalised triphasic and slow waves	3/1	↕ CTX on day 4, diazepam, phenytoin	3
3b	F 12	CPD	100 mg/kg	Confused and unresponsive	NCSE	2/1	↕ CTX on day 2, diazepam	3
4	F 60	AKI (creatinine 114–177, CKD-EPI 28–48)	2	Apathy and somnolence	Generalised triphasic waves	4/3	↕ CTX on day 4	4
5	F 72	HD	1	Lethargy, choreoathetosis of upper and lower limbs	Diffuse delta-theta slow waves with a slow background	3/1	↕ CTX on day 4	5
6	M 76	HD	2	‘Feeling strange’, disorientation, unclear speech, choreoathetosis	–	7/2	↕ CTX on day 9	5
7	M 76	HD	2	Slipped behind his bed, choreoathetosis	–	6/2	↕ CTX on day 8	5
8	F 80	HD	4 (days 1–7), 2 (days 8–18)	‘Behaved as if intoxicated with alcohol’, disorientation, choreoathetosis of extremities	–	5/12	Reduction of CTX dose (4–2 g/24 hours) on day 7, ↕ CTX on day 18	5
9	M 8	Normal (creatinine 52.8)	1	Apathy, somnolence, one episode of myoclonic jerks	–	3/3	↕ CTX on day 3	6
10	F 65	AKI with start of HD during hospitalisation, background CKD (usual creatinine 149.6, CKD-EPI 33)	2	Stupor, generalised myoclonic jerks	Generalised slowing with superimposed bursts of sharp waves, slow wave activity	5/2	↕ CTX on day 7	7
11	F 37	CPD	2	Agitation, paranoia, visual hallucinations	Background moderate diffuse non-specific slowing without epileptogenic activity	3/1.5	↕ CTX on day 3	8
12	M 56	HD	4 (days 1–7), 2 (days 8–15)	Disorientation, agitation, GCS 9, involuntary facial and tongue movements	Bursts of generalised, high-voltage slow-wave activity	7/5	↕ CTX on day 14	9
13	M 72	HD	4 (days 1–7), 2 (days 8–10)	Delirium, GCS 11, myoclonic movements of lower limbs, bilateral Babinski	Diffuse slow-wave activity	8/6	Haloperidol on day 8—improved symptoms. ↕ CTX on day 11	10
14	F 75	HD	2	Agitation, hyperkinesia, confusion, GCS 11	Diffuse slow-wave activity	9/4	↕ CTX on day 9	10
15	F 83	HD	2	Choreoathetosis of extremities	–	3/1	↕ CTX on day 3	11
16	M 76	AKI with HD, background CKD (usual creatinine 123.2 (CKD-EPI 52); peak 290.4 (CKD-EPI 19))	4	Agitation, confusion, GCS 4	Triphasic waves, predominant theta and delta background activity	14/2	Haemodialysis—no effect (day not provided). ↕ CTX (day not provided).	12
17	F 86	HD	1 (days 1–3), 2 (days 3–13)	Decreased level of consciousness, myoclonic jerks	Generalised triphasic waves	13/4	↕ CTX on day 13	13
18	F 70	AKI (creatinine 538 (CKD-EPI 7))	4	Encephalopathy, myoclonus	Severe slowing triphasic waves	3/unspecified	↕ CTX (day not provided)	14
19	F 80	Normal (creatinine 71, CKD-EPI 74))	2.5	Encephalopathy	Moderate slowing triphasic waves	2/unspecified	↕ CTX (day not provided)	14
20	M 73	HD	2	Involuntary movements of limbs, superior and inferior limbs, disorientation, incomprehensible speech, orofacial dyskinesia	Intermittent generalised background slowing	4/4	↕ CTX on day 3 (last scheduled dose)	15
21	F 74	HD	2	Choreoathetosis of limbs, decreased level of consciousness, myoclonus of limbs	Diffuse delta waves, triphasic waves and sharp waves	6/10	↕ CTX on day 6	16
22	M 64	Continuous HD on day 5 for anuric renal failure, intermittent HD from day 13, baseline CKD (MDRD 37).	4	Slowed thinking, acute encephalopathy without focal findings	Toxic encephalopathy	23/3	↕ CTX on day 23	17
23	F 78	HD	1	Drowsiness, GCS 6 without focal findings	Generalised slowing waves	9/6	↕ CTX on day 10	18
24	M 78	HD	2	Trouble of consciousness, GCS 5, myoclonic leg jerks	–	11/1	↕ CTX on day 11, HP on day 11	19
25	M 38	HD	2	Delirium, myoclonic leg jerks, GCS 3	Triphasic waves	4/3	Haloperidol 5 mg (no effect), ↕ CTX on day 5, HP on day 5	19
26	M 84	AKI with background CKD (eGFR 19.9 (day 1), 48.5 (day 14))	2	Trouble of consciousness, partial tonic clonic seizures (face and left upper extremity)	–	8/1	↕ CTX on day 8 (last scheduled dose). Propofol on day 9	20
27	F 73	HD	1	Confused conversations, agitation, hyperkinesia	Generalised slowing waves	4/3	↕ CTX on day 4	21
28	F mid-80s	Normal (creatinine 73, CKD-EPI 64)	2	Fluctuating disorientation, aphasia, loss of contact, myoclonic jerks, chorea-like tongue movements	Generalised discharges with triphasic morphology	4/3	↕ CTX on day 7	Our case

↕, discontinue; AKI, acute kidney injury; CKD, chronic kidney disease; CKD-EPI, Chronic Kidney Disease Epidemiology Collaboration ; CPD, chronic peritoneal dialysis; CTX, ceftriaxone; F, female; GCS, Glasgow Coma Scale; GFR, glomerular filtration rate; HD, haemodialysis; HP, haemoperfusion; M, male; MDRD, modification of diet in renal disease GFR; NCSE, non-convulsive status epilepticus; ref., reference.

Please note that we decided to exclude one case report because of possible confounding factors not detailed in the report^26^ and one case series because of radically different patient descriptions in the written text and the supplied chart.^27^

The mechanism of cephalosporin neurotoxicity is not completely clear but seems to involve a decrease of γ-aminobutyric acid release from nerve terminals and subsequent increase of excitatory neurotransmission.^29–31^ From a pharmacokinetic point of view, the metabolism of ceftriaxone is negligible and it is eliminated renally (55% of the drug) and through the biliary pathway (45%). In patients with mild to moderate renal impairment, changes in the regular dosage regimens are not required, provided liver function is not impaired. In cases of creatinine clearance less than 10 mL/min periodic monitoring of serum ceftriaxone concentrations is recommended, especially if combined with hepatic insufficiency.^1^ A prolonged half-life of elimination is observed in older patients (7.3±1.6 hours in patients aged 18–49 years, 8.3±2.2 hours in patients aged 50–74 years and 14.2±2.9 hours in patients aged 75–92 years).^1^ The age-associated changes in half-life appear to result from changes in systemic clearance since the renal and hepatic functions were normal in all these patients.^1^ Also, the pharmacokinetics of total plasma ceftriaxone are non-linear: the drug is known for a saturable plasma protein binding within the therapeutic range of up to 200 µg/mL with stable free fraction levels at 5%–10% and significantly higher levels of the free drug in supratherapeutic doses (free fraction at 42% at a plasma level of 653 µg/mL).^1^

In the case of CNS effects, the free portion of the drug is the most important to consider, seeing as in the presence of an intact blood–brain barrier, the binding proteins, in particular albumin and globulins, pass the barrier only to a small degree.^17 32 33^

The clinical presentation is not specific and can be easily mistaken for delirium, with most patients being drowsy, having a decreased level of consciousness or agitation. While aimlessly picking at objects and plucking at the air can be associated with delirium^34^, the astute clinician can pick up on chorea-like movements or myoclonic jerks more compatible with encephalopathy.

The diagnosis is mostly one of exclusion, with most reports mentioning a search for electrolyte abnormalities, brain imaging and often a lumbar puncture in a search for secondary causes. The EEG might reveal diffuse slow waves, generalised periodic discharges with triphasic morphology or, in rarer and more severe cases, signs of non-convulsive status epilepticus. Serum and CSF ceftriaxone dosing can also be a helpful diagnostic modality. The normal serum trough level in healthy volunteers receiving 2 g/24 hours has been shown to be 13–15 µg/mL^35^ and the maximum CSF levels after a single 2 g dose in patients with non-inflamed meninges are reported to be 0.18–1.04 µg/mL.^36^ In the reviewed literature, in patients presenting with severe neurological symptoms, the levels were significantly higher than the normal range when measured, with plasma levels varying between 27.9 and 472 µg/mL^10 13 16–19 21^ and the CSF levels being in the range of 5.9–27.9 µg/mL.^13 17–19 21^

The basis of treatment is discontinuing ceftriaxone. Benzodiazepines might be helpful for transient symptom control or in the case of non-convulsive status epilepticus.^2 3^ While ceftriaxone is not removed through haemodialysis^1^, haemoperfusion might hasten elimination and therefore recovery.^19^ In affected patients ceftriaxone readministration should be avoided as it can lead to a new onset of encephalopathy.^3^

Having reviewed the basic pharmacology and available literature, we can assume that the risk factors for developing a ceftriaxone-induced encephalopathy are older age (especially over 75 years old), higher dosing, severely impaired renal or hepatic functions (especially if combined) and undernourishment or decompensated cirrhosis (low binding protein levels leading to a higher fraction of free ceftriaxone).

Our case is notable because of the absence of these factors, except for age. It is of note that the only other published detailed case report of an adult patient having ceftriaxone-induced encephalopathy and normal renal function consists of an 80-year-old woman, receiving a higher than usual ceftriaxone dose for pneumonia (2.5 g/day), who also had a subacute head injury with cerebral contusions.^14^

Our case shows that ceftriaxone-induced encephalopathy should be considered as a differential diagnosis of impaired mental status, even in patients without renal failure.

Looking back at our patient with hindsight, we can also observe how an anchoring bias on parainfectious delirium led to continued ceftriaxone administration, a late consideration of a drug-induced encephalopathy in the context of absent risk factors and a prolonged hospitalisation because of secondary drug effects.

**Learning points**

- Ceftriaxone encephalopathy is rare, possibly under-recognised, and occurs mostly in older patients with severe renal impairment.
- The diagnosis is mostly clinical and made after the exclusion of other probable causes.
- Atypical movements, ceftriaxone dosing and EEG can help orientate the clinician, but the key is to consider the diagnosis, even if the risk factors are lacking.
